# Evidence-Based Alternative Therapies that “Touch the Heart”

**DOI:** 10.36660/abc.20190719

**Published:** 2019-12

**Authors:** Anderson Donelli da Silveira, Ricardo Stein

**Affiliations:** 1 Hospital de Clinicas de Porto Alegre, Porto Alegre, RS - Brazil; 2 Programa de Pós Graduação em Cardiologia e Ciências Cardiovasculares, Universidade Federal do Rio Grande do Sul, Porto Alegre, RS - Brazil; 3 Vitta Centro de Bem Estar Físico, Porto Alegre, RS - Brazil

**Keywords:** Cardiovascular Diseases, Meditation, Relaxation, Yoga, Tai Chi, Sense of Humor, Laughter Therapy

## Introduction

Subcutaneous implantable defibrillator, bioabsorbable polymer stents, transcatheter aortic valve implant for certain patients with severe aortic stenosis, as well as the use of novel anticoagulants, are tangible examples of therapeutic advances in Cardiology in the end of this second decade of the 21^st^ century. In parallel, despite their infinitely lower financial support and prominence in scientific journals and Cardiology Congresses, the non-pharmacological interventions defined as “alternative” have also been tested with the purpose of improving important outcomes in these patients. In Cardiology, studies also use Meditation, Tai Chi (TC), Yoga and even Laughter Therapy as forms of treatment. In this context, this clinical updating has two objectives: 1) To provide the reader of the Arquivos Brasileiros de Cardiologia (ABC cardiol) with access to information on the alternative therapies aforementioned; 2) To enable them, if they are interested, to use these therapies in their everyday professional life; 3) To show that Brazilian researchers have been publishing articles involving some of these alternative therapies in journals like the Arquivos Brasileiros de Cardiologia, as well as in international high impact journals.

### Preamble - Meditation, TC and Yoga

These are ancient oriental practices which, over the last decades, have become more popular and have spread throughout the West. All of them share the fact that they can be found in ancient texts and scriptures, with their foundations often representing an intersection of “sacred” and science. These activities share the integration between body and mind, aiming, in addition to physical and physiological benefits, at changing the perspective of the world in search for greater happiness, quality of life and inner peace. There has been a marked increase in the number of publications on this issue over the last decades, but there are still few well-designed observational studies and randomized clinical trials (RCTs), with no potential bias or conflict of interest, contemplating these three practices. Some of them are detailed below.

### Meditation

It is a practice whose origins reach back to more than 5,000 years and, in spite of being often associated with Buddhism and Hinduism, is present in most religious doctrines, including the three great monotheistic religions (Christianity, Judaism and Islam). The word meditation includes several practices with similar principles like techniques based on Buddhism (*zazen*, *shamatha and vipassana*), Yoga (Raja Yoga meditation), transcendental meditation, mindfulness and even compassion meditation (Tibetan Buddhism). An adequate definition of the technique and the procedures is very important to replicate results and, its absence, is a major methodology problem of several trials that used meditation and have already been published.

Studies report a modest effect of meditation on blood pressure (BP ) decrease, in response to stress, anxiety and smoking cessation.^1^ The effect on BP is small, with a meta-analysis of 19 studies, showing a reduction of 4 to 5 mmHg in systolic pressure and 2 to 4 mmHg in diastolic pressure.^2^ Numerous studies in health and ill populations have explored the effects of meditation in psychological and psychosocial results. It is worth to highlight that most of them report some improvement in perceived stress levels, humor, anxiety, depression, quality of sleeP or overall well-being.^3^

An analysis carried out by the Health Research and Quality Agency, restricted to RCTs and with active control groups, concluded, with low level of evidence, that meditation and mindfulness programs showed modest improvements in stress, anxiety and negative affect.^4^ On the other hand, a grouP of researchers from the Clinics Hospital of Sao Paulo assessed, through an RCT, the practice of meditation compared with a control grouP in patients with heart failure (HF).^5^ A decrease in sympathetic activation was observed in these individuals, as well as improvements in quality of life and increased respiratory efficacy measured by the VE/VCO_2_ slope ratio.

Based on the data aforementioned, it is possible to note some benefits of meditation for patients at high risk or with already established cardiovascular disease. Nevertheless, several gaps still need to be fulfilled, such as the potential effect of meditation on the endothelial function, on heart rate variability (HRV), as well as on primary and secondary prevention of cardiovascular diseases.

### Tai Chi

It is a Chinese martial art with its origins in Chinese traditional medicine and in Taoism. The slow and rhythmic movements aim at interconnecting the movements of the upper and lower limbs synchronously, moving smoothly, continuously and with no breaks and searching for stillness inside the movement. Although the movements of TC are slow and seem to be easy to perform, they work, for many patients, as a type of structured physical exercise.

Studies have demonstrated the beneficial effects of its practice, both in terms of physical and mental aspects, particularly: stress, depression and anxiety reduction; increased functional capacity; BP reduction and improvement of lipid profile.^6,7^ Furthermore, studies in elderly patients have shown a significant improvement in balance, reduced risk of falling, decreased muscle and joint pain, improvement in osteoporosis and even improved or maintained cognitive performance.^6^

Due to a scientific gaP in assessing the impact of TC practice on patients with coronary artery disease - CAD,^8^ the Exercise Cardiology Research GrouP of the Hospital de Clínicas de Porto Alegre studied the effect of this practice on 61 post-AMI patients.^9^ They were randomized for practicing TC for 60 minutes, three times a week or for stretching exercises (control grouP ). After 12 weeks of training, the grouP who practiced TC showed significant improvement in peak oxygen consumption (14% = 3.1 mL.kg^-1^.min^-1^), whereas the stretching grouP did not show any improvements. Similar effects were found in some studies with HF patients, showing that this practice can impact positively on functional capacity increment.

### Yoga

The last of the three oriental practices mentioned is the one which has the largest body of evidence in favor of potential benefits, be it due to a greater volume of studies or to the fact that the practice is more widespread in the West. This practice dates back to 1,500 years B.C. and is present in all Hindu holy books (Vedas, Bhagavad Gita and Upanishads). The word Yoga derives from Sanskrit and means union, and its practice is divided into three components, namely: the *asanas*, which are the different yogic postures; the *pranayamas*, which are breathing exercises and the *dhyanas*, which are basically meditative practices. Different studies have shown the physiological benefits of its practice, among which are metabolic (BP reduction, improvement in lipid and glycemic profile), anti-inflammatory (decrease in C-reactive protein and cytokines), immunological (enhanced CD4 T lymphocytes and telomerase activity), neuroendocrine (decreased cortisol, adrenaline and aldosterone) and autonomic (increased HRV and improved baroreflex sensitivity) effects.^10,11^

The evidences also indicate an increase in oxygen consumption and strength in patients with HF, decreased angina and increased functional capacity in individuals with CAD, as well as reduction of atrial fibrillation symptoms.^10^ A systematic review and meta-analysis assessed the effects of Yoga on some cardiovascular risk factors, and found a mean reduction of 5 mmHg in systolic and diastolic pressures, body mass and body mass index reduction, LDL and triglycerides reduction, as well as increased HDL cholesterol levels.^11^ More recently, a grouP of researchers from different institutions of Rio Grande do Sul carried out an RCT enrolling patients with HF and preserved ejection fraction. The authors compared the effects of Yoga combined with breathing techniques with a control grouP , in accordance with a recently published protocol.^12^ Positive effects were found on inspiratory muscle force and autonomic modulation assessed by HRV in the grouP exposed to the intervention (non-published data).

### Laughter therapy

Laughter is more than a visual and vocal behavior, it is always accompanied by a series of physiological changes, including spasmodic contractions of skeletal muscles, increased heart rate due to catecholamine release and hyperventilation with increased residual air exchange, leading to increased oxygen saturation.^13^ The study of the effect of laughter and humor and its psychological and physiological impacts on the human body is called Gelotology. The first experiments in this area were carried out in the 30s, and assessed the effect of laughter on muscle tone and the respiratory mechanism in laughter. The increase in the number of studies in this area is based on the assumption that, if bad humor is harmful for the cardiovascular system, good humor (laughter therapy) and its physical changes might be beneficial.

One of the few studies that have used Laughter Therapy in unhealthy individuals was performed by Tan et al.^14^ In this experiment, 48 diabetic patients with recent AMI were divided into two groups. The 24 patients in the experimental grouP were assigned to view a humor video for 30 minutes daily, as an adjunct to standard therapy. After a 1-year follow-uP , the authors observed a significant reduction in BP compared with the patients from the control grouP .Furthermore, patients exposed to comedy had fewer episodes of arrhythmias, less use of nitroglycerin for angina and lower incidence of recurrent MI (only two vs. ten cases in the control grouP ).

Finally, an RCT is in progress which aims at assessing hemodynamic biochemical responses of patients with stable CAD undergoing Laughter Therapy.^15^ In this trial, patients of both sexes, aged ≥ 18 years, monitored regularly in a university hospital in the South region of Brazil, are being allocated to an intervention grouP (who will watch a 30-minute comedy film) or to a control grouP (who will watch a 30-minute neutral documentary). It is expected that some results will already be available by 2020.

## Conclusion

We live at a time when Cardiology accelerates towards new technology, when, for instance, artificial intelligence emerges as a true “partner” of physicians. At the same time, ancient traditions such as Meditation, TC and Yoga, in addition to something as delightful as laughter, have been tested and may be used in the management of patients with different cardiomyopathies. Even though these therapies do not present a very robust body of evidence and are usually supported by small efficacy studies, they are simple, safe and low-cost therapeutic alternatives which, in addition to improving quality of life, can positively influence the physiological and biochemical parameters of these individuals. Finally, there is a perspective that, as the number of adherents of these practices increases, larger and better-designed studies will be carried out, which may establish the real role of these practices, whether in prevention or treatment of cardiovascular diseases.
